# Selective vulnerability of the intermediate retinal capillary plexus precedes retinal ganglion cell loss in ocular hypertension

**DOI:** 10.3389/fncel.2022.1073786

**Published:** 2022-12-05

**Authors:** Priyamvada M. Pitale, Guofu Shen, Rohini R. Sigireddi, Maria Polo-Prieto, Yong H. Park, Solomon E. Gibson, Peter D. Westenskow, Roomasa Channa, Benjamin J. Frankfort

**Affiliations:** ^1^Department of Ophthalmology, Baylor College of Medicine, Houston, TX, United States; ^2^Department of Neuroscience, Baylor College of Medicine, Houston, TX, United States; ^3^Department of Ophthalmology and Visual Sciences, University of Wisconsin-Madison, Madison, WI, United States

**Keywords:** ocular hypertension, capillary remodeling, hypoxia, glaucoma, neurovascular unit (NVU)

## Abstract

**Introduction:** Glaucoma, a disease of retinal ganglion cell (RGC) injury and potentially devastating vision loss, is associated with both ocular hypertension (OHT) and reduced ocular blood flow. However, the relationship between OHT and retinal capillary architecture is not well understood. In this project, we studied microvasculature damage in mice exposed to mild levels of induced OHT.

**Methods:** Mild OHT was induced with the microbead model for 2 weeks. At this time point, some retinas were immunostained with CD31 (endothelium), Collagen IV (basement membrane), and RBPMS (RGCs) for z-stack confocal microscopy. We processed these confocal images to distinguish the three retinal capillary plexi (superficial, intermediate, and deep). We manually counted RGC density, analyzed vascular complexity, and identified topographical and spatial vascular features of the retinal capillaries using a combination of novel manual and automated workflows. Other retinas were dissociated and immunopanned to isolate RGCs and amacrine cells (ACs) for hypoxia gene array analysis.

**Results:** RGC counts were normal but there was decreased overall retinal capillary complexity. This reduced complexity could be explained by abnormalities in the intermediate retinal capillary plexus (IRCP) that spared the other plexi. Capillary junction density, vessel length, and vascular area were all significantly reduced, and the number of acellular capillaries was dramatically increased. ACs, which share a neurovascular unit (NVU) with the IRCP, displayed a marked increase in the relative expression of many hypoxia-related genes compared to RGCs from the same preparations.

**Discussion:** We have discovered a rapidly occurring, IRCP-specific, OHT-induced vascular phenotype that precedes RGC loss. AC/IRCP NVU dysfunction may be a mechanistic link for early vascular remodeling in glaucoma.

## Introduction

Glaucoma is chronic, progressive optic nerve degeneration characterized by the irreversible loss of retinal ganglion cells (RGCs) and damage to the optic nerve head (ONH). It is one of the leading causes of blindness with an expected global disease burden of over 100 million patients (Tham et al., 2014). Traditionally, the pathogenesis of glaucoma has been explained by two clinical models: the mechanical and vascular theories of glaucoma. Elevated intraocular pressure (IOP), or ocular hypertension (OHT), drives the mechanical theory, while microvascular deficits and reduced blood flow drive the vascular theory. However, the two theories may not be mutually exclusive. For example, rodent experimental glaucoma models have shown that reduced blood flow, oxidative stress, and the resultant endoplasmic reticulum (ER) stress activation due to the local hypoxic conditions in the ONH and inner retina lead to neurodegeneration (Doh et al., 2010; Chidlow et al., 2017; Syc-Mazurek et al., 2017; Kasetti et al., 2020). Moreover, clinical studies in glaucoma patients using ocular coherence tomography angiography (OCTA) report that transient IOP fluctuations may cause hypoxic injury which leads to microvascular changes in both the retina and ONH (Yarmohammadi et al., 2016; Jia et al., 2017; Liu et al., 2019; Tepelus et al., 2019). Despite these intriguing correlations among IOP, glaucoma, and retinal vasculature, a direct cause and effect relationship between IOP and vascular abnormalities has not been established.

The complex vasculature of the inner retina is a three-tiered network consisting of the superficial retinal capillary plexus (SRCP), intermediate retinal capillary plexus (IRCP), and deep retinal capillary plexus (DRCP). The SRCP supplies the retinal nerve fiber layer (RNFL), RGC somas, and the dendrites of ON-RGCs in the inner plexiform layer (IPL). The IRCP maintains the dendrites of the OFF-RGCs in the IPL and amacrine cells (ACs) in the inner nuclear layer (INL). The DRCP supports bipolar cells (BPs) and horizontal cells (HCs) in the outer plexiform layer (OPL; Usui et al., 2015; Nian et al., 2021). Each plexus supports a neurovascular unit (NVU) of neurons, pericytes, endothelial cells, and astrocytes, the components of which differ according to the depth of the retina and the associated capillary plexus (Usui et al., 2015; Nian et al., 2021).

Even though RGCs are the primary cells affected in glaucoma, a range of electrophysiological, anatomic, and transcriptional studies have shown that early dysfunction is also seen in ACs, OFF-RGCs, and their synapses, often before RGC soma loss (Dijk et al., 2004; Kielczewski et al., 2005; Crish et al., 2010; Sappington et al., 2010; Gunn et al., 2011; Frankfort et al., 2013; Pang et al., 2015; Akopian et al., 2019; Park et al., 2019; Tao et al., 2019). The timing and mechanism of these events, especially in the setting of normal RGC soma numbers, remain unclear. In this manuscript, we utilize an experimental glaucoma model of mild OHT in mice which has only minimal RGC loss (7.4%) after 6 weeks of IOP elevation (Frankfort et al., 2013) and no obvious RGC loss after 2 weeks of IOP elevation (Tao et al., 2019). With this model, we test the hypothesis that vascular changes occur rapidly in the anatomic region of highest neuronal and dendritic susceptibility to elevated IOP—the distal IPL and INL, where OFF-RGC dendrites and ACs reside, respectively. Furthermore, we determine if these changes precede RGC loss.

## Methods and Materials

### Animal use and induction of experimental glaucoma

All animal experiments were approved by the Institutional Animal Care and Use Committee of Baylor College of Medicine and conducted in adherence with the ARVO Statement for the use of animals in ophthalmic and vision research and the NIH guide for the use of laboratory animals. C57BL6J (Wild type, WT) mice were purchased from Jackson laboratories (stock no. 000664). Twelve-week-old mice were injected with polystyrene beads or saline followed by sodium hyaluronate in the anterior chamber of one eye as previously described and observed for 2 weeks (Frankfort et al., 2013; Tao et al., 2020a). Briefly, mice were given an intraperitoneal injection of a stock anesthetic of ketamine 37.5 mg/ml, xylazine 1.9 mg/ml, and acepromazine 0.37 mg/ml. The experimental eye was dilated with 1% tropicamide and 2.5% phenylephrine and the cornea was topically anesthetized with 0.5% proparacaine hydrochloride. A mix of polystyrene beads in a total volume of 1.5 μl (6 μm diameter blue polystyrene beads, cat#15715-5; and 1 μm diameter yellow polystyrene beads, cat#15713-15; Polysciences, Inc., Warrington, PA) followed by 3 μl of sodium hyaluronate (cat#571182 Provisc; Alcon Laboratories, Ft. Worth, TX) was injected through corneal perforation created with a 30 g needle. The non-injected eye was the intra-animal control for all experiments. IOP measurements were performed under isoflurane anesthesia (after 8 min of sedation). IOP values were obtained three times a week in the morning to confirm persistent IOP elevation in all mice.

### Immunohistochemistry

Retinal dissection was performed following established protocols in the Frankfort lab (Frankfort et al., 2013; Tao et al., 2020b). Dissected whole-mount retinas were fixed with 4% paraformaldehyde for 1 h at room temperature and blocked with 10% donkey serum overnight. Retinas were then incubated in primary antibodies [Collagen IV (EMD Millipore cat#AB756p; 1:300), CD31 (BD bioscience cat#550274; 1:50), and RBPMS (Phospho solutions cat#1832; 1:250)] diluted with 3% donkey serum for 5 days at 4°C, followed by overnight incubation at 4°C in secondary antibodies [Alexa fluor 647 donkey anti-rabbit (Jackson Immuno Research Labs cat# 711-605-152; 1:300), Cy3 donkey anti-rat (Jackson Immuno Research Labs cat#712-165-153; 1:300), Alexa fluor 488 donkey anti-guinea pig (Jackson Immuno Research Labs cat#706-545-148; 1:300), and Hoechst 33,342 nuclear staining (Invitrogen cat#H3570; 1:1,000)] diluted with 3% donkey serum.

### Image processing and analysis

Z stack images of flat-mounted retinas were acquired with laser confocal microscopy (Zeiss LSM 800). 10× images of the entire retina were collected for Collagen IV (COL IV) and CD31. 20× images were collected for COL IV, CD31, RBPMS, and Hoechst in four quadrants of the retina at a position 750 μm from the optic nerve (Frankfort et al., 2013). RBPMS positive RGCs were manually counted by an investigator who was blinded to the experimental conditions using the ImageJ cell counter plugin. Sholl analysis of 10× CD31 immunostained images was performed to determine differences in the complexity of the entire retinal vasculature. For the Sholl analysis, we calculated the number of vasculature intersections with concentric rings of increasing radii (50 μm increments) from the optic nerve head up to 1,500 μm using the ImageJ Sholl plugin. An estimate of the density of intersections in a full ring [*D_ring_* (*r*, *δr*)] with radius *r* and with radial thickness *δr* was obtained by dividing the experimentally observed number of interactions (*I_obs_*) by the area of the ring (*A_ring_*; van Pelt et al., 2014). In our analysis, a radial thickness of *δr* = 1 μm was used for each ring.


$$D_{\text{ring}}=\frac{I_{\text{obs}}}{A_{\text{ring}}}=\frac{I_{\text{obs}}}{2\pi r\delta r}$$

Additional processing of 20× magnification images was performed with ImageJ to separate stacked images for each retinal plexus for COL IV and CD31. These images were analyzed with NIH open-source AngioTool software using a custom workflow (Supplementary Figure 1). This software computes topographical vascular features such as capillary branch points (junctions) and spatial dimensions such as vascular length and vascular coverage percentage area, to provide semi-automated quantification which limits investigator bias (Zudaire et al., 2011). Acellular capillary density was manually counted in COL IV immunostained retinas at 20× magnification.

### Amacrine cell (AC) and retinal ganglion cell (RGC) isolation and PCR array

ACs and RGCs were isolated using a previously described immunopanning technique (Park et al., 2019, 2020). Positive panning plates for CD15+ (anti-SSEA-1 #BD560079; BD Pharmingen, San 136 Jose, CA) and CD57+ (anti-HNK-1/N-CAM#C6680-100TST; Sigma Aldrich, St. Louis, MO) specific for ACs, and the positive panning plate for Thy1.2 (#MCA02R; Bio-Rad Antibodies, Hercules, CA) specific for RGCs were washed to remove any non-adherent retinal cells. Adherent ACs and RGCs were dissociated using trypsin and further processed to isolate RNA. For the PCR array, total RNA isolation was performed according to the manufacturer’s protocol using the TRIzol/spin column-based nucleic acid extraction kit (Direct-Zol; #R2050, Zymo Research, Irvine, CA, USA). Following cDNA construction, hypoxia-related gene expression was measured using the RT^2^ Profiler PCR Arrays: The Mouse Hypoxia Signaling Pathway (GeneGlobe ID-PAMM-032Z, Qiagen). Gene expression was normalized to housekeeping genes.

### Statistical analysis

Data are presented throughout as mean ± SEM. A comparison of cumulative IOP over time was performed by calculating the area under the curve (AUC) using the trapezoidal method. The final average IOP was performed using paired *t*-tests (two-sided). For Sholl analysis, the number of intersections was plotted against the radius and the AUC was calculated. Retinal vasculature data were analyzed using paired *t*-tests (two-sided). The PCR array for gene expression was expressed as a heatmap using the log transformation values, with *p* = 0.05 as the cutoff for significance. All the analyses were conducted using Prism (GraphPad, La Jolla, CA). The thresholds for statistical significance are represented as *p < 0.05; **p < 0.01; ***p < 0.001).

## Results

### Mild OHT for 2 weeks does not cause RGC loss

**Figure 1:**
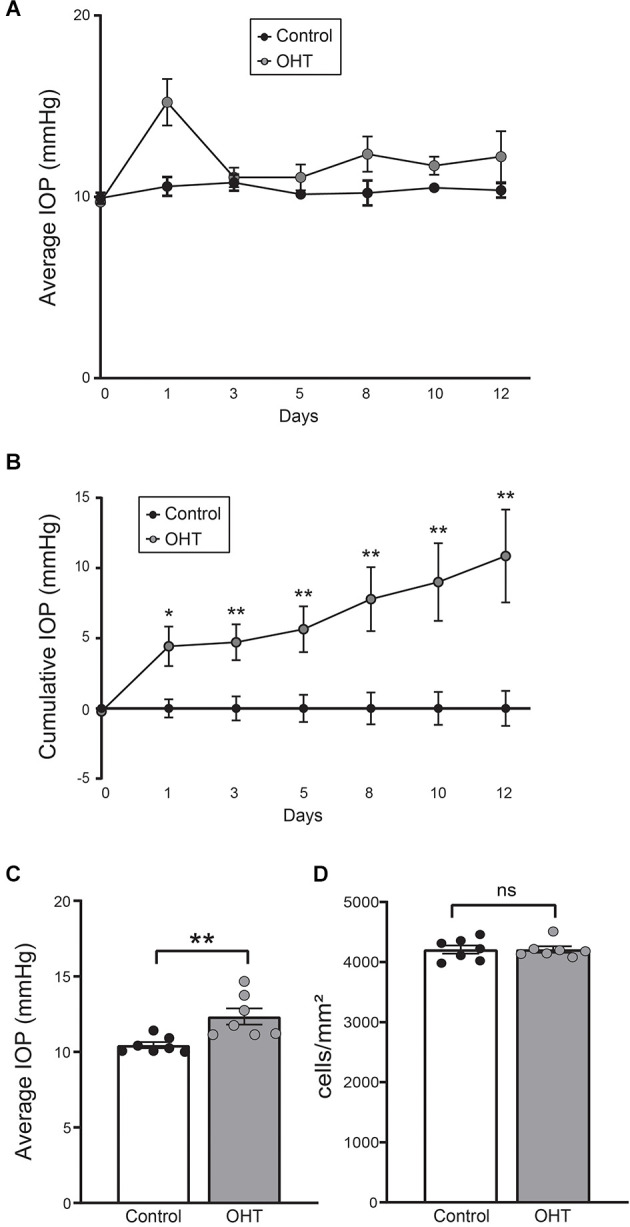
**(A)** Average daily intraocular pressure (IOP) over the 2-week long experiment. **(B)** Cumulative IOP normalized to baseline. There was a consistent elevation in cumulative IOP throughout the study (paired *t*-test, *n* = 7; four females, three males). **(C)** Average IOP of all measurements. Bead injected eyes showed a significant increase in IOP compared to normal eyes (paired *t*-test, *n* = 7; **p < 0.01). **(D)** RGC density was equivalent between control and ocular hypertension (OHT) eyes (ns, nonsignificant). *p < 0.05, **p < 0.01.

Eyes injected with polystyrene beads demonstrated mild OHT with IOP elevation of 18.15% ± 4.38% (*p* < 0.01) compared to the fellow, non-injected eyes (Figures 1A–C). RGC density (cells/mm^2^) was determined using RBPMS (RGC marker) immunostaining and was similar between IOP elevated and control eyes. This suggests that no RGC loss occurred at this level and duration of IOP elevation (Figure 1D; Supplementary Figure 2).

### Mild OHT for 2 weeks causes reduced retinal capillary complexity

**Figure 2:**
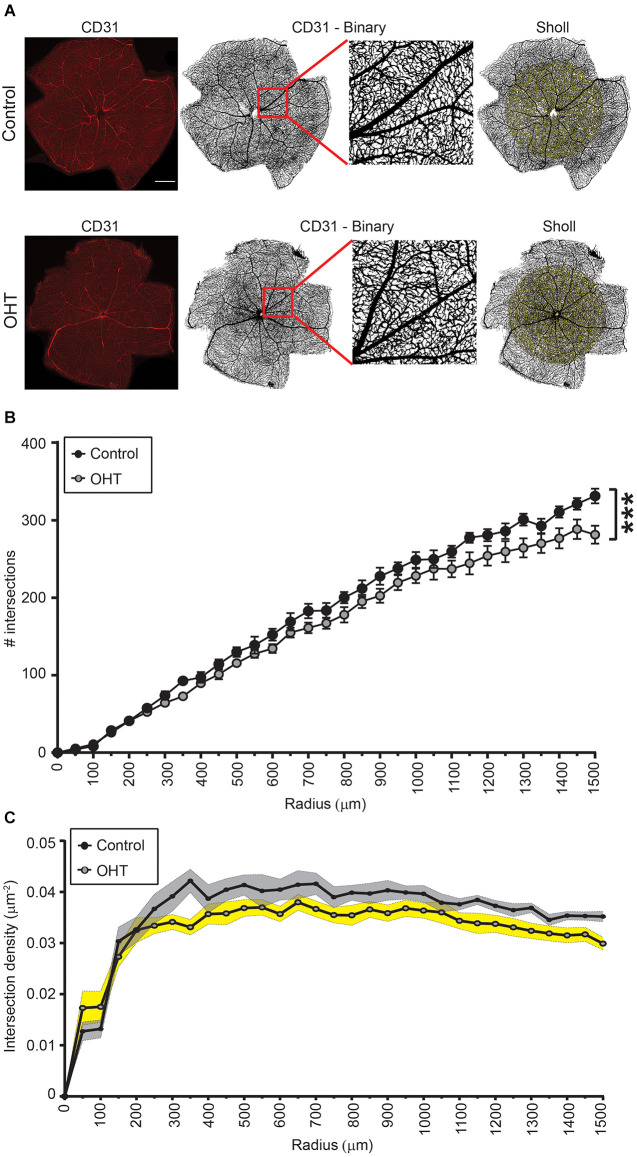
**(A)** Representative images of CD31 immunostained retinas for control (top) and OHT (bottom) eyes. Left panels. Original image (scale bar = 500 μm). Middle two panels. Binary image of the same retinas with inset showing increased magnification. Right panels. Retina showing Sholl analysis patterns (right panels). **(B,C)** Sholl analysis for radii ranging from 0 to 1,500 μm. **(B)** The total number of intersections was significantly reduced in OHT retinas (AUC; ***p < 0.001). **(C)** Graph showing the total number of intersections as a function of the retinal explant area (intersection density) for both control and OHT eyes.

We next assessed retinal capillary complexity using immunostaining with CD31 (endothelial cell marker) at the same time point, 2 weeks after IOP elevation, using Sholl analysis (Figure 2A). The AUC of the number of intersections was reduced for OHT (241,104 ± 4,512) compared to control (267,314 ± 4,028) eyes, indicating an overall reduction of retinal complexity (*p* < 0.001; Figure 2B). In control eyes, the relative vascular complexity showed a gradual increase from the optic nerve head to the mid-retina which then stabilized to a plateau phase from the mid-retina to the periphery, as expected (Figure 2C; Giannakaki-Zimmermann et al., 2016; Campbell et al., 2017; Lavia et al., 2020). In OHT eyes, our data demonstrated that retinal complexity started to diminish prior to the mid-retina (~300 μm), with persistently reduced complexity all the way to the periphery (Figure 2C). Therefore, 2 weeks of mild OHT significantly reduced the complexity of the retinal microvasculature despite no obvious effect on RGC density.

### Mild OHT for 2 weeks preferentially impacts the intermediate retinal capillary plexus

**Figure 3:**
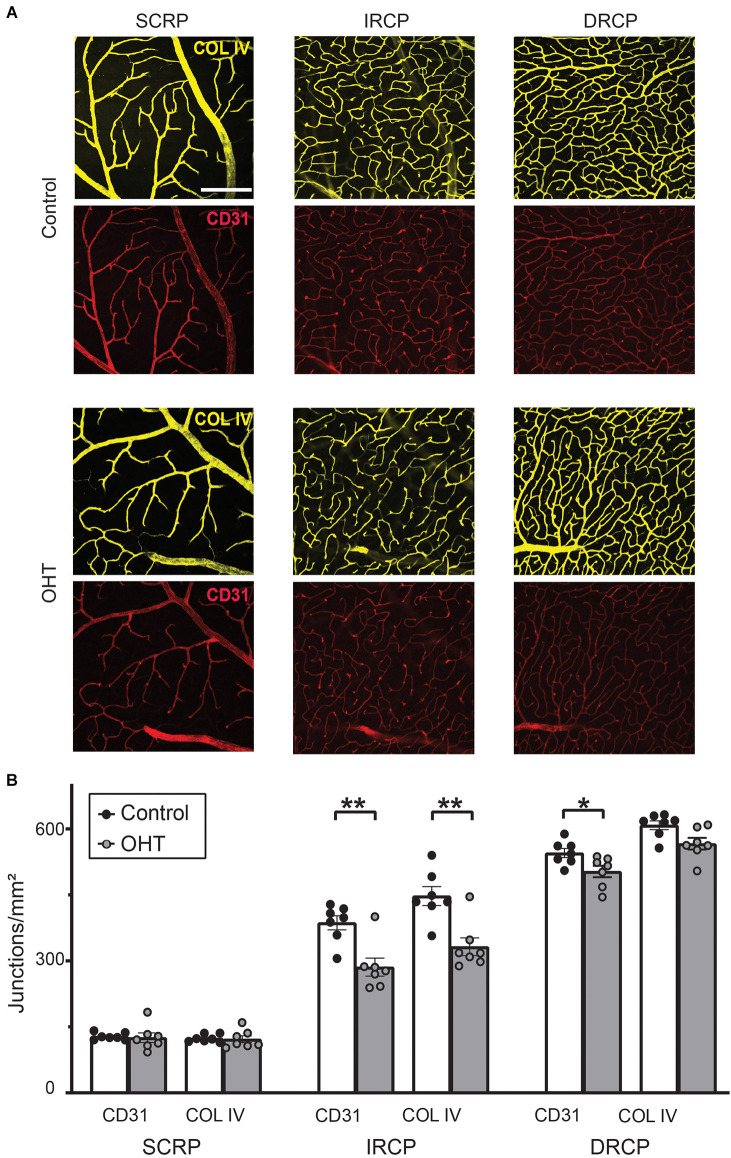
**(A)** Representative images for Collagen IV and CD31 immunostained retinas for superficial retinal capillary plexus (SRCP), intermediate retinal capillary plexus (IRCP), and deep retinal capillary plexus (DRCP; scale bar = 200 μm). **(B)** Junction density (number of junctions/mm^2^) for CD31 and Collagen IV immunostained retinas. No significant changes were observed for the SRCP. There was a significant reduction in junction density for both CD31 and Collagen IV in the IRCP for OHT retinas (paired *t*-test; **p < 0.01). Junction density was reduced for CD31 but not Collagen IV for OHT retinas in the DRCP (paired *t*-test; *p < 0.05).

To assess plexus-specific phenotypes after mild IOP elevation, we developed a novel image analysis workflow using ImageJ and AngioTool software (NIH open-source, Supplementary Figure 1 and “Methods” Section; Zudaire et al., 2011). Stacked images of each capillary plexus immunostained for both COL IV (basement membrane marker) and CD31 (endothelial marker, Figure 3A) were analyzed to semi-automatically quantify the vascular anatomical features and spatial distribution of the retinal capillaries. After 2 weeks of mild OHT, there was a significant reduction in the number of capillary junctions/mm^2^ (junction density) for both CD31 (26% ± 5.91%, *p* ≤ 0.01) and COL IV (26% ± 5.77%, *p* ≤ 0.01) in the IRCP for OHT eyes (Figure 3B). Similarly, we saw an 8% decrease in the junction density in the DRCP for CD31 but not COL IV. There was no reduction in the junction density in SRCP for either CD31 or COL IV. These data suggest that the IRCP is the most susceptible plexus to mild IOP elevation.

**Figure 4:**
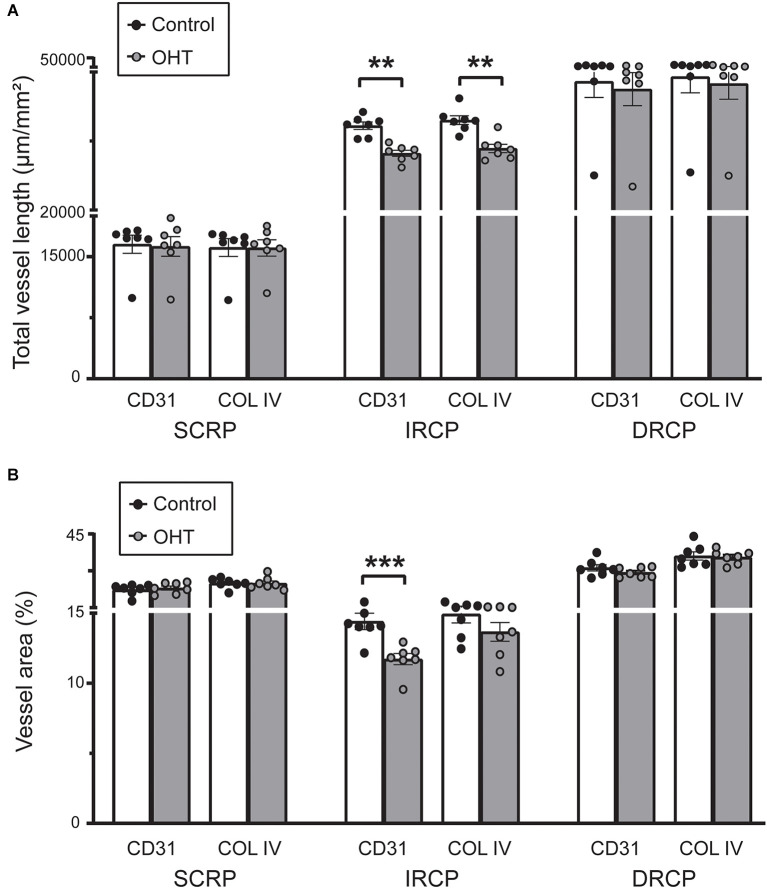
**(A)** Total capillary vessel length (μm/mm^2^) in CD31 and Collagen IV immunostained retinas. The total length was reduced for both CD31 and Collagen IV in the IRCP for OHT retinas (paired *t*-test; **p < 0.01). No significant changes were seen in the SRCP and DRCP. **(B)** Percentage of the retina covered by vessels (vessel area %) for CD31 and Collagen IV immunostained retinas. The vessel area percentage was decreased for CD31 but not for Collagen IV in the IRCP for OHT retinas (paired *t*-test; ***p < 0.001). No significant changes were seen in the SRCP or DRCP.

Next, we studied spatial vascular features in the capillary plexus. The total capillary vessel length/mm^2^ (vessel length) was reduced for both CD31 (12% ± 2.04%, *p* ≤ 0.01) and COL IV (12% ± 2.55%, *p* ≤ 0.001) in the IRCP after 2 weeks of mild OHT (Figure 4A). There was also a significant decrease in the vessel area percentage for CD31 (19% ± 2.09%, *p* ≤ 0.01) but not COL IV in the IRCP (Figure 4B). Neither of these spatial vascular features were significantly changed in the SRCP or DRCP.

**Figure 5:**
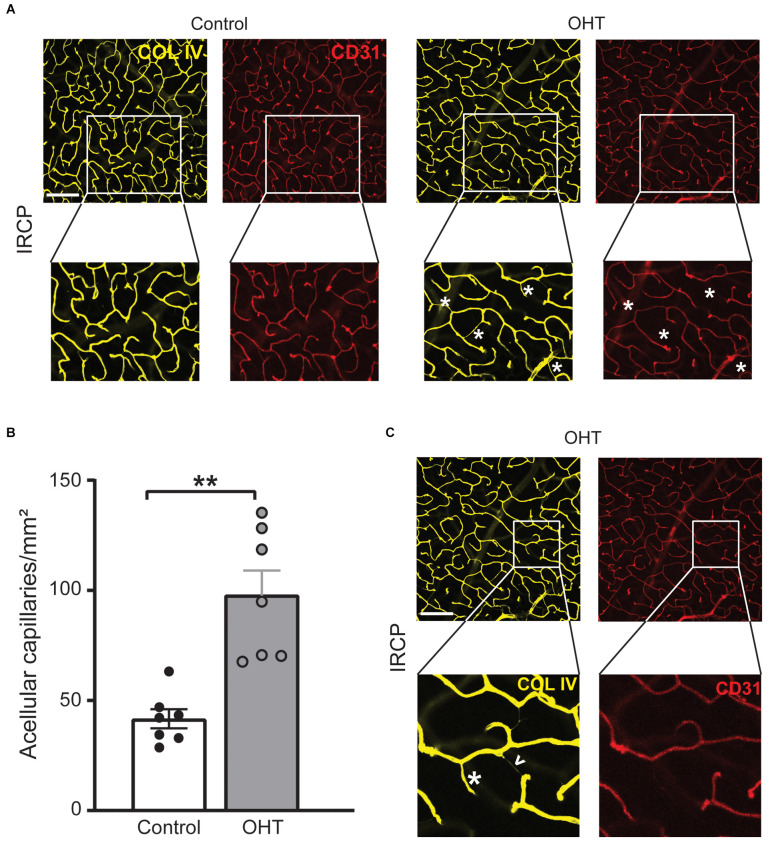
**(A)** Representative IRCP images for Collagen IV and CD31 immunostained retinas (top; scale bar = 200 μm) with zoom for additional detail (bottom). Magnified images show examples of acellular capillaries (ghost vessels) marked with white asterisks. **(B)** Acellular capillary density (acellular capillaries/mm^2^) was greatly increased in the IRCP for OHT retinas (paired *t*-test; **p < 0.01). **(C)** Different magnified region of the same representative images (**A**, top) highlights the difference between acellular capillaries (white asterisks) and inter-pericyte tunneling nanotubes (IP-TNTs; white arrowhead).

Since COL IV identifies the basement membrane and CD31 marks endothelial cells they normally exist in a precise spatial relationship in which COL IV surrounds CD31. After injury, however, this relationship can be disturbed as endothelial cells retract, leaving behind the basement membrane. These acellular capillaries or “ghost vessels” represent capillary dropout (Baluk et al., 2003; Veenstra et al., 2015). Thus, to further assess IRCP-specific changes to spatial vascular features we interrogated the relationship between COL IV and CD31. Acellular capillary density was significantly increased (Figures 5A,B) after 2 weeks of mild OHT (134.9% ± 44.6%, *p* ≤ 0.01). Importantly, acellular capillaries can be differentiated from the inter pericyte tunneling nanotubes (IP-TNTs) as the latter are much thinner and are associated with a pericyte proximally or distally (Figure 5C; Alarcon-Martinez et al., 2020).

Taken together, these data suggest that capillary dropout and remodeling in the IRCP are among the earliest plexus-specific, identifiable and quantifiable responses to mild IOP elevation.

### Mild OHT for 2 weeks causes increased hypoxia in amacrine cells (ACs)

**Figure 6:**
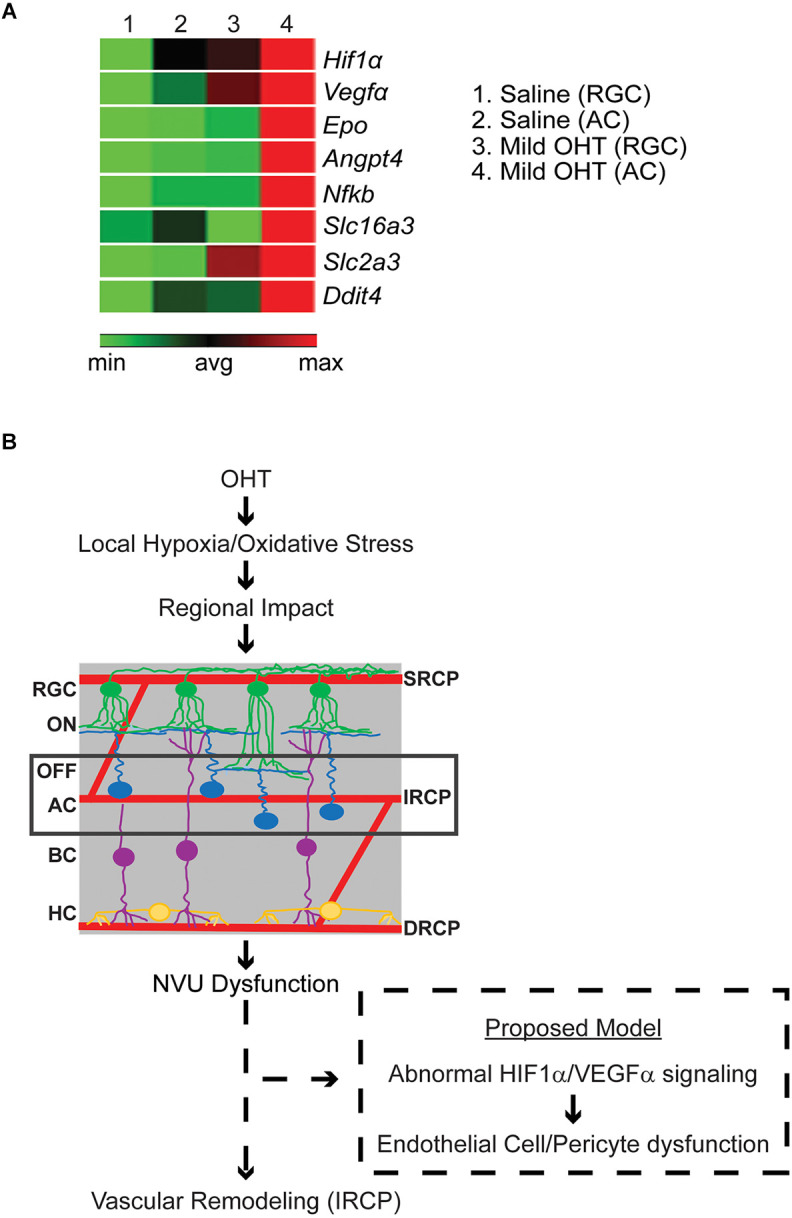
**(A)** Relative gene expression of hypoxia pathway genes in pooled immunopanned amacrine cells (ACs) and retinal ganglion cells (RGCs) from mild OHT and saline control eyes. RT Profiler was used to quantify the relative expression of multiple hypoxia genes. After IOP elevation, ACs (column 4), but not RGCs (column 3), show a relative increase in expression (red) of the HIF1α-target genes *VEGFα, Epo, Angpt4, Nfkb, Slc16a3, Slc2a3,* and *Ddit4* (*n* = 3 biological replicates). **(B)** Potential model of OHT impact on the retinal vasculature. With mild OHT, the resulting hypoxia and oxidative stress lead to microvascular remodeling in the retinal capillary plexi and disrupted physiology of retinal cells. The earliest impacted retinal cell and retinal capillary plexus are ACs and the IRCP, respectively. It is likely that neurovascular unit (NVU) dysfunction results in abnormal HIF1α/VEGFα signaling to cause AC-Endothelial cell-pericyte miscommunication and resultant IRCP remodeling.

Since regulation of the IRCP involves the interplay of signals from several NVU members including HIF1α-dependent signaling from ACs (Usui et al., 2015), we further investigated whether ACs displayed evidence of local hypoxia. To do so, in a parallel experiment, after 2 weeks of bead- (OHT, average IOP increase = 1.53 mmHg) or saline-injection (control, no IOP increase), ACs and RGCs were isolated using immunopanning (Park et al., 2019, 2020). Extracted RNA was used to quantify gene transcription from a series of hypoxia related genes (RT Profiler; Qiagen). We found a relative increase in the expression of many hypoxia-dependent genes in ACs>>RGCs from mild OHT eyes. Interestingly, this included several HIF1α target genes related to vascular remodeling, angiogenesis, inflammation, neurodegeneration, and metabolite transport (Figure 6A). Thus, it is likely that local hypoxia in ACs but not RGCs is an early indicator of IOP-induced molecular change.

## Discussion

In this study, we show that mild OHT causes preferential IRCP remodeling and local hypoxia which precedes RGC loss. This novel vascular phenotype has several implications for glaucoma pathogenesis.

The IRCP develops at around p12–p15 as AC synaptic development and resultant increases in energy demand trigger the upward sprouting of the IRCP from the DRCP as directed by a transient gradient of ischemia-driven VEGF. This pathway is again utilized during adulthood as ACs regulate and maintain the IRCP *via* HIF-1α/VEGF-dependent mechanisms (Voinescu et al., 2009; Usui et al., 2015). This signaling likely works through NVUs which consist of ACs, endothelial cells, pericytes, and glia (intermediate plexus NVU, or iNVU; Usui et al., 2015; Nian et al., 2021). Interestingly, the IRCP is the most vulnerable of all three plexi to hypoxia and oxidative stress (Usui et al., 2015). Our data, which show enhanced sensitivity of the IRCP to IOP elevation and upregulation of HIF-1α/VEGFα expression in ACs, support and expand these observations. Indeed, multiple studies in experimental glaucoma have found that ACs are among the first neurons to be affected, but the mechanism of their initial injury is unclear (Gunn et al., 2011; Pang et al., 2015; Akopian et al., 2019). Therefore, we propose that early iNVU dysfunction is the linking event between IRCP remodeling and AC dysfunction in experimental glaucoma.

NVU crosstalk among its four cell types is complex (Kugler et al., 2021; Nian et al., 2021). While our data cannot clearly distinguish the absolute first event in iNVU dysfunction, multiple possibilities can explain the combination of IRCP remodeling, AC dysfunction, and AC upregulation of HIF-1α/VEGFα signaling (Figure 6B). Upregulation of HIF-1α/VEGFα signaling is known to cause increased capillary development (Ramakrishnan et al., 2014; Zimna and Kurpisz, 2015). However, since we see the opposite—decreased IRCP capillary complexity—it is unlikely that direct, IOP-induced AC injury is the first event in iNVU dysfunction. Rather, the decreased IRCP complexity that we see may represent vascular stress, and the upregulation of HIF-1α/VEGFα signaling that we see in ACs is therefore more likely to be a compensatory response to local hypoxia (Schulz et al., 2012; Tsuboi et al., 2015). Other NVU components such as pericytes and glial cells also play important roles in retinal capillary development and control capillary blood flow according to neuronal demand (Zhang and Stone, 1997; Bergers and Song, 2005; Wareham and Calkins, 2020). Additional studies will be necessary to distinguish the specific order and location of NVU dysregulation after IOP elevation.

Our data may also explain the observation that OFF-RGCs, and in particular their dendrites, are more susceptible to IOP elevation than ON-RGCs (Della Santina et al., 2013; El-Danaf and Huberman, 2015; Ou et al., 2016; Della Santina and Ou, 2017; Sabharwal et al., 2017). Since the primary blood supply for the OFF stratum of the IPL (dendrites of OFF-RGCs) is the IRCP (Usui et al., 2015; Nian et al., 2021), the reduced local blood flow expected with IOP-induced IRCP capillary retraction is a plausible anatomic explanation. We observed subtle changes in the expression of HIF-1α targets in RGCs and these may represent OFF-RGCs. However, additional studies will be required to confirm this.

Interestingly, the impact of OHT on the capillary plexi may differ according to the level and/or duration of IOP elevation. For example, a previous study from our lab found that acutely elevated IOP to high levels led to delayed capillary injury to both the SRCP and the IRCP (Tao et al., 2020b). The very high levels of IOP in that study (>50 mmHg) suggest that distinct IOP injury thresholds may exist for the IRCP (low IOP) and the SRCP (high IOP). This interpretation is consistent with other threshold effects that have been seen in induced glaucoma models (Tao et al., 2019, 2020a) as well as the differential effects of acute (HIF-1α related) and chronic (HIF-2α related) hypoxia on cellular injury (Tezel and Wax, 2004; Mowat et al., 2010).

This study is limited to observations of changes in the structural anatomy of the retinal vasculature and does not assess the underlying vascular physiology of the IRCP. Nevertheless, the findings in this manuscript may help explain the pathogenesis of several findings in patients with glaucoma. First, conditions associated with abnormal blood circulation (migraines, Reynaud’s phenomenon, systemic hypotension, etc.) are well associated with normal tension glaucoma (Mallick et al., 2016; Chan et al., 2017). Reduced local blood flow or tissue oxygenation at the level of the capillary plexi may provide a pathogenic link. Second, the prevalence of glaucoma increases with age (Allison et al., 2020; Zhang et al., 2021). Similarly, aging reduces vascular plasticity/capillary remodeling (Lahteenvuo and Rosenzweig, 2012; Xu et al., 2017). Our data suggest that inspection of the IRCP in the elderly population might hold diagnostic and predictive value for glaucoma, as it does for other retinal diseases (Iafe et al., 2016; Ye et al., 2020; Gao et al., 2022). Third, small fluctuations in IOP cause microvascular injury and are associated with additional RGC loss even when average IOPs are equivalent (Nouri-Mahdavi et al., 2004; Caprioli and Coleman, 2008; Nita and Grzybowski, 2016; Wareham and Calkins, 2020). Thus, small variations in IOP may create a susceptible environment for neuronal injury in normal tension or treated glaucoma.

## Data Availability Statement

The raw data supporting the conclusions of this article will be made available by the authors, without undue reservation.

## Ethics Statement

The animal study was reviewed and approved by Baylor College of Medicine IACUC.

## Author Contributions

PP conducted confocal imaging, generated the image analysis workflow, analyzed data, prepared figures, and wrote the primary manuscript draft. GS conducted immunohistochemistry and recorded IOP. RS performed pilot studies and developed imaging procedures. MP-P assisted in data analysis and managed animal breeding. YP and SG conducted cell isolation, RNA analysis experiments, and analyzed data. PW provided protocols and conceptualized research hypotheses. RC assisted with vascular hypotheses and clinical correlation. BF obtained funding, conceptualized research hypotheses, and supervised the entire project. PP and BF edited the final draft manuscript and reviewed final figures. All authors contributed to the article and approved the submitted version.

## Funding

This work was supported by National Eye Institute (NEI) Grants R01 EY025601 (BF) and P30 EY002520 (Baylor College of Medicine) and an unrestricted grant from Research to Prevent Blindness to the Cullen Eye Institute at Baylor College of Medicine.
