# Supplementary material for: Selective vulnerability of the intermediate retinal capillary plexus precedes retinal ganglion cell loss in ocular hypertension
Source: Front Cell Neurosci. 2022 Dec 5;16:1073786. doi: 10.3389/fncel.2022.1073786 (PMC9760765; doi:10.3389/fncel.2022.1073786)

Supplementary Material

Supplementary Figure. 1. Schematic for the image analysis workflow (top). **A.** Four confocal Z-stacks (mid-retina) were acquired 750µm from the optic nerve (circle diameter = 1500 µm). ImageJ was used to separate the individual plexi and AngioTool software was used to semi-automatically quantify the topographical and spatial capillary features. **B.** Example IRCP stacked COL IV image. **C.** Automatic vessel definition by AngioTool. **D.** Manual vessel refinement based on vessel diameter with AngioTool. **E.** Final automatic analysis of the image for branch points, vessel length, and vessel area. **E'.** Vessel lengths are shown in green (white circle) and junctions (branch points) are shown in red (white arrow).

Supplementary Figure. 2. Representative images of RBPMS immunohistochemistry for control and OHT eyes. (Scale bar = 200µm).

Sup Fig. 1

SEMI-AUTOMATED IMAGE ANALYSIS WORKFLOW

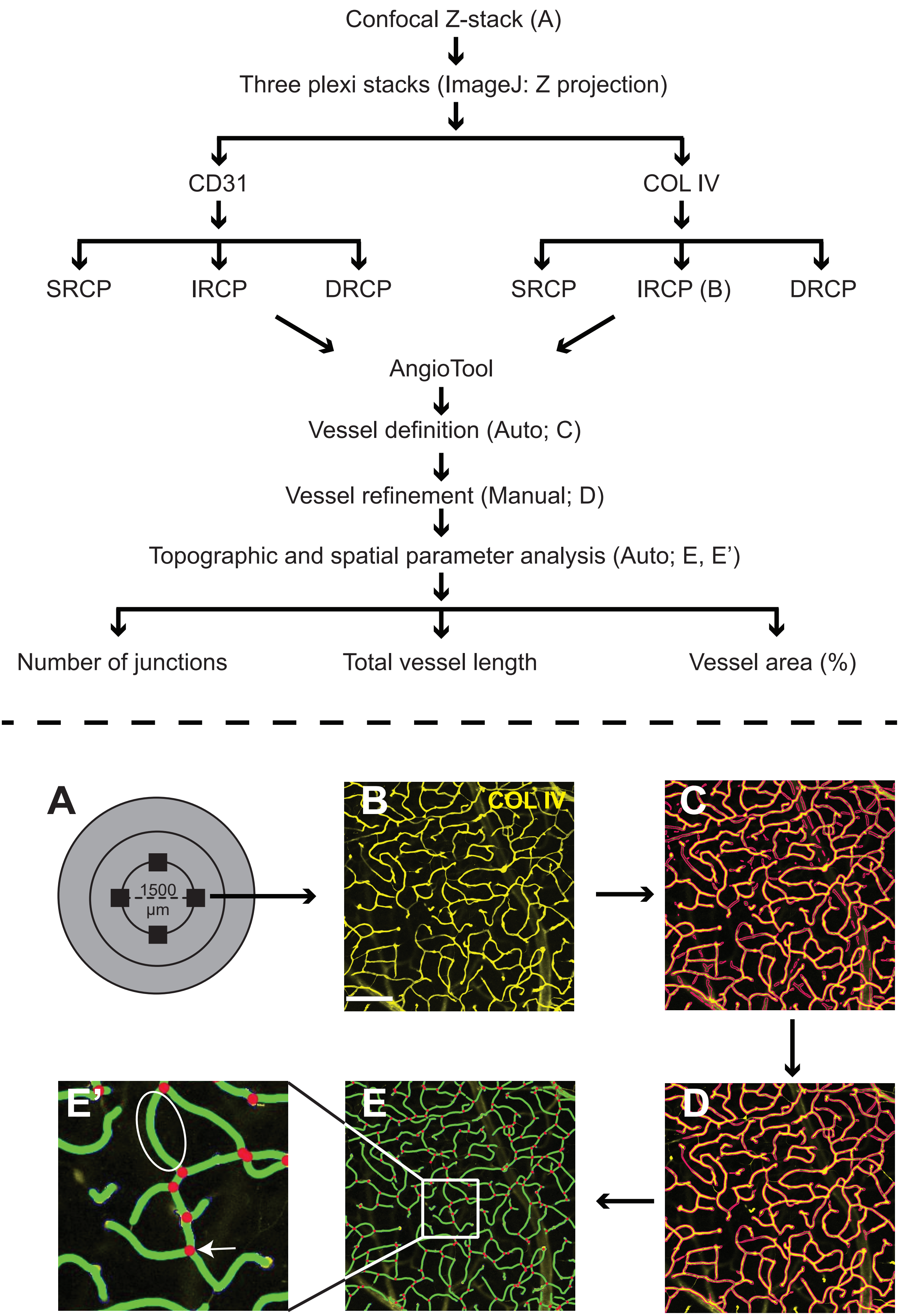

Sup Fig. 2

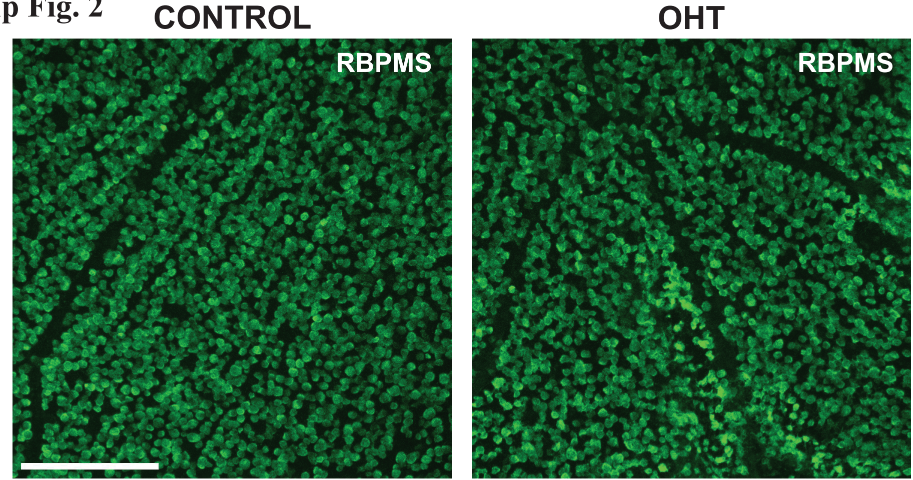

Supplement: Supplementary file 1 [file Data_Sheet_1.PDF]
